# Rare malignant peripheral nerve sheath tumor of vagus nerve: A case report

**DOI:** 10.1016/j.ijscr.2023.107940

**Published:** 2023-02-18

**Authors:** Aleksandra Borovika, Renārs Deksnis, Jānis Zariņš, Sergejs Isajevs

**Affiliations:** a Riga Stradiņš University, Faculty of Residency, Riga, Latvia; b Riga East University Hospital Latvian Centre of Oncology, Department of Head and Neck Surgery, Riga, Latvia; c Riga Stradiņš University, Department of Otolaryngology, Riga, Latvia; d Department of Hand and Plastic Surgery, Microsurgery Centre of Latvia, Riga, Latvia; e Baltic Biomaterials Centre of Excellence, Headquarters at Riga Technical University, Riga, Latvia; f Department of Pathology, Faculty of Medicine, University of Latvia, Riga, Latvia; g Riga East University Hospital, Centre of Pathology, Riga, Latvia; h Hospital of Traumatology and Orthopaedics, Department of Microbiology and Pathology, Riga, Latvia

**Keywords:** Case report, MPNST, Vagal nerve, Nerve reconstruction, Nerve graft

## Abstract

**Introduction:**

MPNST is a rare type of malignancy classified as malignant soft tissue sarcoma. One-fourth to one-half of MPNST arise in patients with neurofibromatosis type 1 (NF1) and generally involves major nerve trunks of proximal extremities and body, rarely head and neck region. Aggressive nature of the disease shows poor overall prognosis, where treatment modalities are also limited.

**Presentation of case:**

62-year-old otherwise healthy female underwent radical surgical treatment due to the mass of the right side of the neck. Preoperative MRI studies showed well defined partly cystic and visually malignant neoplasm of the carotid sheath in upper third of the neck. Well-defined tumor of the right vagus nerve was detected during the surgery and was excised with safe and radical margins. Further histological study confirmed MPNST diagnosis. Defect of the vagus nerve was reconstructed with a nerve grafts to maintain and improve patients quality of the life. Adjuvant radiotherapy was appointed. At one year follow-up period no evidence of disease recurrence was found. Nevertheless, patient reported significant improvement of functionality and less vagus nerve impairment symptoms.

**Discussion:**

In this article we discuss main epidemiological data of MPNST as well as distinction of our clinical case peculiarities from data mentioned in literature.

**Conclusion:**

MPNST are described as aggressive neoplasms with unfavorable short and long-term prognosis. Early diagnosis and radical surgical intervention not only improve patient prognosis but also allow to use additional treatment options to improve patients survival and quality of the life even in case of MPNST.

## Highlights

• Malignant peripheral nerve sheath tumor (MPNST) is a rare type of malignancy particularly in head and neck region.
• Vagus nerve is an unusual site of MPNST localization.
• Early diagnosis and radical excision of vagal nerve MPNST improve prognosis and allow to use additional treatment options.

## 1 Introduction

MPNST is a rare locally aggressive neoplasm with incidence of 0.001 % in general population and overall poor prognosis. MPNST are classified as malignant soft tissue sarcomas. One-fourth to one-half of MPNST arise in the setting with NF1. MPNST commonly involve major nerve trunks. Most frequent site of localization are proximal extremities - 35 to 60 % of all MPNST, followed by trunk - up to 35 %. Recent studies have demonstrated that head and neck region is a rare site of MPNST and accounts up to 12 % [1], [2], [3].

In this case report we present a 62-year-old otherwise healthy female with recent history of right-sided neck mass. Examination and radiologic imaging indicated a well-defined partly cystic visually malignant neoplasm of carotid sheath in upper 1/3 of the right side of the neck. Intra-operative findings revealed well-defined mass of the right side vagus nerve which was excised with a safe and radical margins. Histological examination confirmed MPNST diagnosis. The resection lines were negative for tumor tissue and local invasion was not found. In early postoperative period patient experienced disturbing vagus nerve damage symptoms such as voice hoarseness and cough. Due to patient's decreased quality of the life one stage reconstruction with a sural nerve graft surgery was performed. Adjuvant radiotherapy was appointed to reduce local recurrence. At one year follow-up period no evidence of disease recurrence was found, patient reported significant improvement of functionality and less vagus nerve impairment symptoms.

The work has been reported in line with the SCARE criteria [4].

## 2 Case report

**Fig. 1:**
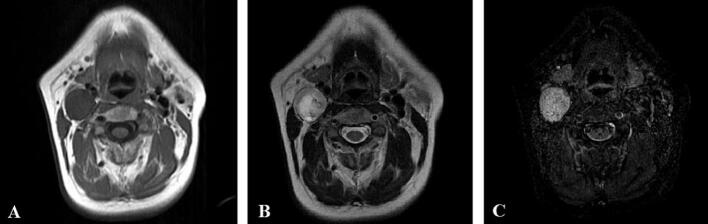
MRI scan in axial plane indicating neoplasm of carotid sheath of the right side of neck. A- T1, B- T2, C- T2 STIR sequences.

**Fig. 2:**
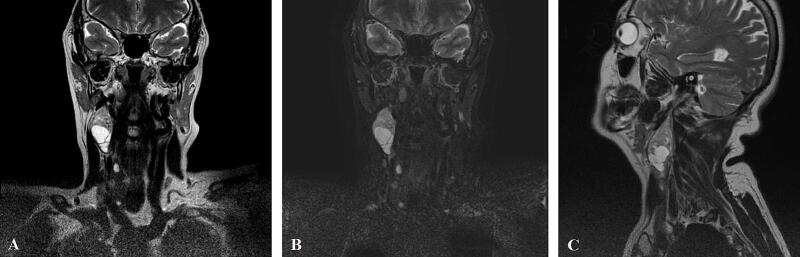
MRI scan indicating neoplasm of carotid sheath of the right side of neck. A- coronal plane T2 sequence, B- coronal plane T2 STIR sequence, C- sagittal plane T2 sequence.

62-year-old female admitted to Riga East University Hospital, Oncology Centre of Latvia, Department of head and neck surgery with complaints about right-sided neck mass over the past three months that gradually increased in the size. Physical examination of the patient revealed semi-mobile neck mass in upper 1/3 of the right side of neck about 2.5 cm in diameter. Further radiological examinations as ultrasound and contrast-enhanced magnetic resonance imaging (MRI) scan indicated well-defined partly cystic visually malignant neoplasm of carotid sheath 2.3 × 2.5 × 4.3 cm in upper 1/3 of the right side of neck with no signs of cervical lymphadenopathy (Fig. 1, Fig. 2). Other physical and instrumental examination of the patient was completely normal. Patient did not have any other significant accompanying medical conditions as well no physical or anamnestic data of NF1.

**Fig. 3:**
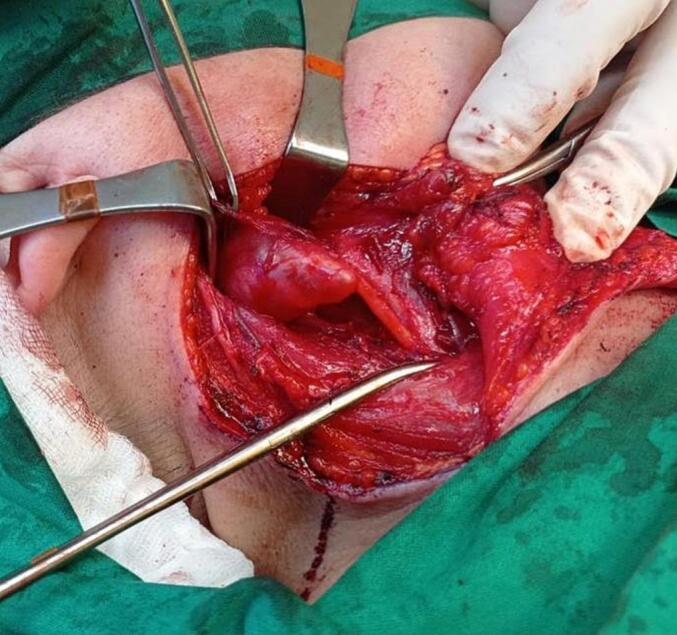
Intraoperative finding- solid and well-defined mass of the right side vagus nerve.

**Fig. 4:**
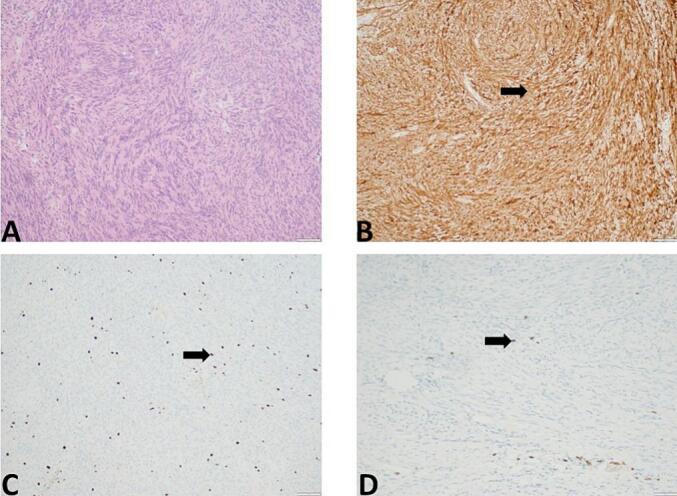
Representative photomicrograph demonstrated MPNST. A-hematoxylin eosin staing method, magnification ×100, scale bar-50 μm. B-S-100 expression, immunohistochemical staining method, magnification ×100, scale bar-50 μm. C- Ki-67 expression, immunohistochemical staining method, magnification ×200, scale bar-100 μm; D- SOX-10 expression, immunohistochemical staining method, magnification ×200, scale bar-100 μm.

Surgery was performed. Excision of solid and well-defined mass of the right side vagus nerve (Fig. 3) with safe and radical margins was done. About 6 cm long vagus nerve defect was identified. Excised mass was sent for frozen section pathology. Frozen section result was suspicious to Schwannoma. For this reason no additional surgical modalities (e.g. nerve reconstruction) was done during the surgery. Pathological examination revealed the tumor of the size of 4x3x2cm. The tumor consisted from spindle cells with hyperchromatic, thin, wavy, or focally buckled nuclei, some epithelioid cell, with fascicular growth pattern with focal necrosis up to 5 %, moderate mitotic activity up to 10–12 mitosis per 10 high powered field at magnification ×400, with hypocellular and hypercellular areas with perivascular accentuation, hemosiderin deposition, without lymphovascular invasion. The immunohistochemistry demonstrated that tumor cells were focally positive for S100 and SOX-10, Ki-67 index was 15 %, CD99 showed focal positivity. The tumor cells were negative for CD34, ERG, CD117 and DOG- (Fig. 4).

In first postoperative day patient complained about voice hoarseness and throat discomfort. Laryngoscopy revealed partial paresis of right vocal cord. Chest X ray showed normal state of diaphragmatic cupola. Soon patient complaints intensified, additional symptoms as spontaneous non-productive cough appeared. Spontaneous cough, especially during the night time and voice hoarseness were the most disturbing symptoms. Patient complained about mild symptoms of dyspnea during physical activity, hiposensitivity of right auricle, periodic belching, nausea and vomiting after cough, heartburn, periodic mild swallowing difficulties.

**Fig. 5:**
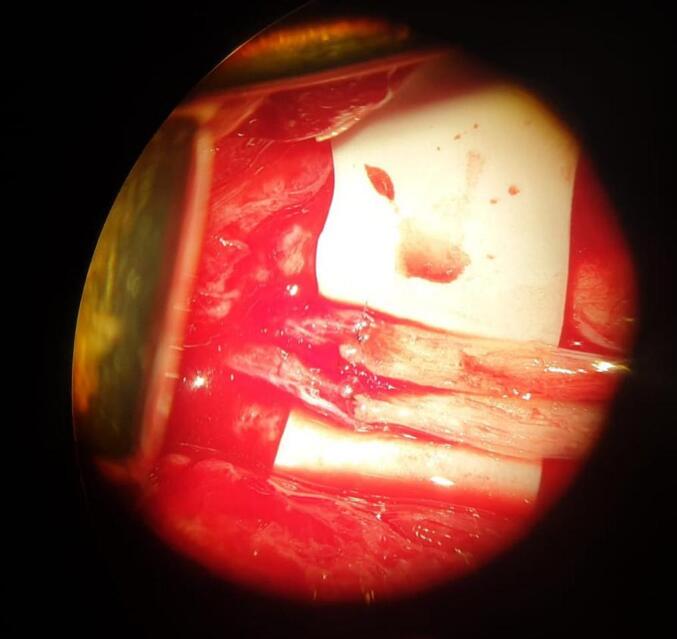
Intraoperative microscopic image - vagus nerve reconstruction with sural nerve grafts.

To improve the quality of the life and restore the functions of the vagus nerve, additaional surgery was planned. Right vagus nerve reconstruction surgery with right sural nerve graft was performed (Fig. 5). Even in the early postoperative period patient noticed decrease of cough episodes. On 24 hospitalization day patient was discharged from the hospital. Subseqently patient received adjuvant thirty 2.0 Gy fractions of stereotactic body radiation therapy of right neck region. Also myofunctional training course was done.

No data of local recurrence or metastases were found during the one year follow-up period.

Patient experienced various symptoms after vagus nerve tumor excision surgery. Voice hoarseness and spontaneous cough patient marked as most disturbing. Right after the reconstructive surgery and on regular follow up visits patient was asked to evaluate vagus nerve impairment symptoms by using visual analogue scale to estimate nerve graft surgery results and evaluate patients quality of the life. On 3-month follow up patient did not noticed any significant improvement of previous symptoms, however at 6 months as well as one year after surgery patient reported significant improvement of main symptoms - cough episodes became less frequent, voice hoarseness was less intensive as well as other symptoms became milder or disappeared at all.

On one-year follow up visit patient filled up Voice Handicap Index with score 20, that describes her voice handicap as mild. Subjectively patient evaluated her voice as strongly disturbed in first post-operative months, moderately disturbed on 6-months follow up and as mildly disturbed on 1-year follow up.

According to above-mentioned information, authors evaluate nerve graft surgery result as positive, with good impact on patient's voice and overall long term quality of the life.

Now patient continues to undergo regular head and neck surgeon check-ups.

Written informed consent was obtained from the patient for publication of this case report and accompanying images. A copy of the written consent is available for review by the Editor-in-Chief of this journal on request.

## 3 Discussion

MPNST is a rare type of peripheral nerve tissue malignancy. Incidence reported in literature is about 0.001 % in general population [1]. MPNST are classified as malignant soft tissue sarcomas and account for approximately 5 % to 10 % of it. MPNST in 25 % to 50 % of cases arise in patients with NF1, 10 to 20 years after radiation therapy and the rest cases being sporadic [2], [3], [8].

According to recent reports, more often MPNST are found in young adults, 20–50 years old with median age of 35, with a male predominance. However in case of sporadic MPNST gender ratio is almost equal. More aggressive course and earlier onset of disease is characteristic to patients with NF1.

Clinically MPNST presents as gradually enlarging painless deep soft tissue mass that is usually located in proximal extremities. Less common site of localization is trunk followed by head and neck region. Most MPNST cases arise from major nerve trunks. In course of disease patients can develop sensory and motor deficiency symptoms such as weakness, paresthesias and projected pain.

Diagnosis is made according to imaging studies and biopsy results. Open biopsy is preferred. MRI is the imaging modality of choice. Size of tumor >5 cm, heterogenity, surrounding oedema and poor-defined tumor margins are highly suspicious for MPNST [5], [6].

Treatment of choice is R0 surgical resection. There is no exact adjuvant radiation treatment strategies of MPNST mentioned in literature because of limited research data avaliable to guide treatment selection. Usually it is advocated for large tumors (>5 cm) to reduce local recurrence [8]. In our case adjuvant radiation therapy was suggested to patient with aim to minimize any risks of local recurrence and improve patient's long term survival because of aggressiveness of the MPNST nature. Still there are not enough data about the effectiveness of adjuvant chemotherapy for patients with MPNST [8].

According to literature prognosis of MPNST is poor. MPNST are defined as high-grade tumors with high recurrence and metastatic potential. It is reported that local recurrences develop in about 40 % of patients within 1 year of the original surgery. Metastases develop in 40 % to 60 % of patients within 12 months of surgery [2], [8]. 5-year disease specific survival rate varies from 15 to 50 % [7], [8]. As main adverse prognostic factor is mentioned large tumor size (>5 cm), as well as presence of metastases, history of local recurrence, truncal location, surgical margin status [2], [3], [8].

Clinical case of our patient is quite unique because the age of onset of disease is indistinctive and differs from data mentioned in literature. Our patient was overthise healthy with no physical or anamnestic data of NF1. Site of tumor origin is rare and absence of local invasion is not typical for MPNST. Due to absence of tumor local spread it was possible to choose additional treatment options - nerve graft surgery - to improve patient life quality. In 3, 6 and 12 month follow-up no data of local recurrence or metastases were found. Improvement of vagus nerve damage symptoms as a result of performed nerve graft surgery was noted.

## 4 Conclusion

MPNST is a rare and challenging clinical condition either for clinician and patient. Even short term prognosis of disease is unfavorable. However early diagnosis with subsequent radical surgical treatment and adjuvant radiation therapy improves patient prognosis at least in short term follow-up and allow to use additional treatment options (e.g. reconstructive surgery) to improve patient's quality of the life.

## Consent

Written informed consent was obtained from the patient for publication of this case report and accompanying images. A copy of the written consent is available for review by the Editor-in-Chief of this journal on request.

## Funding

Not applicable.

## Ethical approval

Case report. No need for ethical approval.

## Author contribution

Aleksandra Borovika: collecting data, writing and editing the article, corresponding.

Renārs Deksnis: surgeon of the patient, editing the article.

Jānis Zariņš: surgeon of the patient, editing the article.

Sergejs Isajevs: Preparing and reporting the pathology images, editing the article.

All authors read and approved the final version of the manuscript.

## Research registration number

Not applicable.

## Guarantor

Renārs Deksnis.

## Declaration of competing interest

Not applicable.
